# Two-Dimensional Mo_*x*_W_1−*x*_S_2_ Graded Alloys: Growth and Optical Properties

**DOI:** 10.1038/s41598-018-31220-z

**Published:** 2018-08-27

**Authors:** Kevin Bogaert, Song Liu, Tao Liu, Na Guo, Chun Zhang, Silvija Gradečak, Slaven Garaj

**Affiliations:** 1 Department of Materials Science and Engineering, Massachusetts Institute of Technology, 77 Massachusetts Avenue, Cambridge, MA 02139 USA; 2 Centre for Advanced 2D Materials, National University of Singapore, 6 Science Drive 2, 117546 Singapore, Singapore; 3 Department of Physics, National University of Singapore, 2 Science Drive 3, 117542 Singapore, Singapore; 4 Department of Chemistry, National University of Singapore, 3 Science Drive 3, 117543 Singapore, Singapore; 5 Low Energy Electronic Systems Interdisciplinary Research Group, Singapore-MIT Alliance in Research and Technology, 1 CREATE Way, 138602 Singapore, Singapore; 6 Department of Biomedical Engineering, National University of Singapore, 9 Engineering Drive 1, 117575 Singapore, Singapore

## Abstract

Two-dimensional (2D) transition metal dichalcogenides can be alloyed by substitution at the metal atom site with negligible effect on lattice strain, but with significant influence on optical and electrical properties. In this work, we establish the relationship between composition and optical properties of the Mo_*x*_W_1−*x*_S_2_ alloy by investigating the effect of continuously-varying composition on photoluminescence intensity. We developed a new process for growth of two-dimensional Mo_*x*_W_1−*x*_S_2_ alloys that span nearly the full composition range along a single crystal, thus avoiding any sample-related heterogeneities. The graded alloy crystals were grown using a diffusion-based chemical vapor deposition (CVD) method that starts by synthesizing a WS_2_ crystal with a graded point defect distribution, followed by Mo alloying in the second stage. We show that point defects promote the diffusion and alloying, as confirmed by Raman and photoluminescence measurements, density functional theory calculations of the reaction path, and observation that no alloying occurs in CVD-treated exfoliated crystals with low defect density. We observe a significant dependence of the optical quantum yield as a function of the alloy composition reaching the maximum intensity for the equicompositional Mo_0.5_W_0.5_S_2_ alloy. Furthermore, we map the growth-induced strain distribution within the alloyed crystals to decouple composition and strain effects on optical properties: at the same composition, we observe significant decrease in quantum yield with induced strain. Our approach is generally applicable to other 2D materials as well as the optimization of other composition-dependent properties within a single crystal.

## Introduction

Two-dimensional (2D) transition metal dichalcogenides (TMDs) are layered semiconducting materials with an array of interesting properties, including a direct bandgap^1,2^, large exciton binding energy^3^, large spin orbit coupling^4^, and a high degree of band structure tunability via surface dopants^5–7^ and alloying^8–26^, which make them desirable for optical, electronic, and energy generation applications^27–33^.

In analogy to conventional semiconductors, substituting various transition metal (M = Mo, W, etc.) and chalcogen (X = S, Se, Te) atoms into the TMD lattice while maintaining an MX_2_ stoichiometry would enable property modulation for specific applications. Whereas spatially uniform TMD alloying offers a choice of material properties within a range given by its pure TMD components, graded TMD alloying – in which the composition gradually varies across the crystal – introduces anisotropic behavior in the bandgap, optical properties and spin-orbit coupling that could be implemented, for example, in future excitonic devices^34^. The previous studies of alloyed TMDs have demonstrated that the photoluminescence (PL) bandgap emission energy is tunable as a function of composition, but most of these studies used discrete crystals with a specific alloy composition. This approach hinders understanding of the intrinsic relationship between PL quantum yield and composition, as it does not account for non-compositional factors between different crystals (*e*.*g*., substrate interactions, strain profile, defect density, etc.) that could influence the optical properties (*e*.*g*., charge transfer doping, band structure modification, creation of trap states, etc.).

To circumvent this limitation, we study optical properties of 2D TMDs with compositional variations within single crystals that were produced using a novel 2-step chemical vapor deposition (CVD) process based on diffusion-mediated growth method. In the first step, WS_2_ crystals are grown via CVD, which are then exposed to MoS_2_ precursors in the second step. The resulting crystals show unexpected compositional variations where Mo atoms diffuse and replace W atoms inside the crystals^17^. In this work – driven by a hypothesis that the alloying is facilitated by point defects – we introduce the graded distribution of point defects into the template WS_2_ crystal by mixing the metal precursor (WO_3_) with NaCl admixture that is known to reduce its vaporization temperature^35^. As a result, in the second CVD step, the diffusion of Mo results in a unique graded alloy structure mirroring the starting defect distribution. By having the ability to tune composition within a single crystal, we demonstrate composition-dependent variations in PL intensity that are intrinsic to the material, and not an artifact of the sample preparation. Moreover, we decouple strain- and composition-related optical properties assessing the role of substrate in future applications of 2D TMDs. Here we focus on Mo_*x*_W_1−*x*_S_2_ alloys, but our synthesis method and the general approach is generally applicable across various TMD platforms.

## Results and Discussion

**Figure 1:**
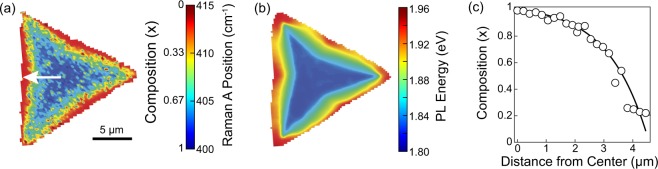
(**a**) Raman position of the A vibrational mode (and thus the composition *x*) of a Mo_*x*_W_1−*x*_S_2_ graded alloy crystal. Scale bar is 5 μm. (**b**) Corresponding PL peak energy map. (**c**) Crystal composition as a function of the position along the arrow shown in (a) determined using the Raman A vibrational mode. The line is a guide for eye.

Details of the synthesis are described in the Methods section. In short, to produce compositionally-graded Mo_*x*_W_1−*x*_S_2_ 2D single crystals, WS_2_ crystals were first grown at 825 °C using WO_3_/NaCl precursor, followed by the deposition of MoS_2_ at 680 °C. Figure 1a shows the Raman A vibrational mode of a representative resulting single crystal and the corresponding optical microscopy and atomic force microscopy (AFM) images are provided in the Supporting Information, Figure S1. It has been demonstrated that the position of the A vibrational mode shifts linearly over the composition range from ~403 cm^−1^ for pure MoS_2_ (*x* = 1) to ~419 cm^−1^ for pure WS_2_ (*x* = 0)^26^. As it can be seen in Fig. 1a, the A mode in our crystals changes gradually from 400 cm^−1^ in the center of the crystal to 415 cm^−1^ toward the crystal edges, thus demonstrating continuously changing Mo_*x*_W_1−*x*_S_2_ composition. The PL peak energy map of the same crystal (Fig. 1b) corroborates the Raman results shown in Fig. 1a. The exciton emission energies of pure MoS_2_ and WS_2_ are 1.82 eV and 1.97 eV, respectively^36^, whereas the measured PL energy range spans from 1.82 eV to 1.95 eV starting from the crystal core toward edges, respectively. An additional example of a crystal demonstrating this behavior is presented in Supporting Figure S2. Taken together, these results indicate that the crystal has a three-fold symmetry with pure MoS_2_ in the core and gradually-shifted composition toward the crystal edges that are composed of almost pure WS_2_. This gradient in composition is quantified in Fig. 1c, calculated from the Raman data presented in Fig. 1a.

This graded spatial variation of composition seen in the final alloyed crystal structure was enabled by engineering a spatially heterogeneous concentration of point defects in the starting WS_2_ crystals. Figure S3 in Supporting Information shows a Raman spectrum and the Raman intensity ratio map that compares LA(M) and A vibrational modes of a template WS_2_ crystal grown using the NaCl-based CVD method. It has been previously shown that the LA(M)/A Raman intensity ratio is proportional to the inverse square of the distance between point defects with low-defect crystals having a ratio of 0.10–0.15^37^. WS_2_ crystals grown without NaCl show a uniform ratio ≤0.15;^17^ in contrast, WS_2_ crystals grown using NaCl are more defective with the ratio ranging from 1.05 at the crystal core to 0.80 at the edges. The graded LA(M)/A intensity ratio present in NaCl-assisted WS_2_ crystals is indicative of a graded point defect distribution, with a greater density of defects near the crystal core compared to the exterior. This is likely due to a W-rich atmosphere during early stages of the crystal growth leading to non-stoichiometric crystal growth and formation of sulfur vacancies. This result indicates that the higher density of point defects in the core of the starting WS_2_ crystal enhances Mo diffusion and results in the graded Mo concentration in the resulting alloyed structure.

**Figure 2:**
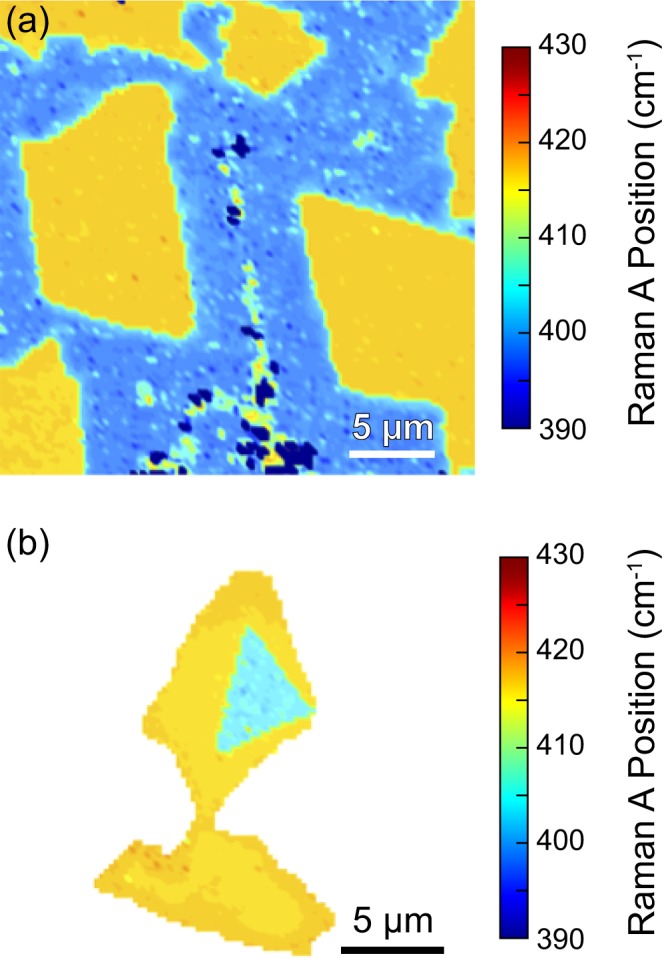
Raman position of the A vibrational mode for (**a**) exfoliated WS_2_ (yellow) followed by CVD-grown MoS_2_ (blue) and (**b**) exfoliated MoS_2_ (blue) followed by CVD-grown WS_2_ (yellow). Both growths result in a lateral heterostructure with a sharp interface and chronological core-shell configuration indicating that no significant amount of diffusion occurred. Scale bars are 5 μm.

To further probe the role of defects in our novel graded alloy synthesis method, we mechanically exfoliated WS_2_ or MoS_2_ template crystals – having a low defect density – and employed second stage CVD growth of the complementary material (Fig. 2). A WS_2_ crystal was first exfoliated and deposited onto a substrate, followed by the standard MoS_2_ CVD deposition. Figure 2a shows the A Raman vibrational mode for the resulting crystal. As evidenced by the sharp interface between the mechanically exfoliated WS_2_ (A position ≈415 cm^−1^) and CVD-grown MoS_2_ (A position ≈405 cm^−1^) regions, no alloying or diffusion has taken place. A comparable sharp interface is observed for crystals with the inverse configuration, consisting of a mechanically exfoliated MoS_2_ core and the surrounding CVD-grown WS_2_ (Fig. 2b).

**Figure 3:**
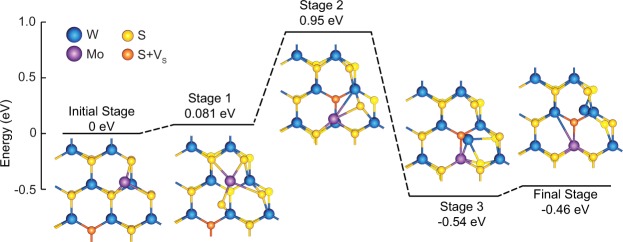
DFT-calculated proposed reaction path and schematics of atomic configurations for inward diffusion of a Mo atom and outward diffusion of a W atom and S vacancy. From the Initial Stage to Stage 2, a Mo adatom and S atom diffuse inward together, resulting in the outward diffusion of a S vacancy. From Stage 2 to the Final Stage, the Mo adatom incorporates into the metal plane of atoms, displacing a W atom, which then diffuses outward through the S vacancy site to the crystal edge. This reaction is exothermic.

To gain microscopic insight into the diffusion process, we performed density functional theory (DFT) modeling and explored possible diffusion-exchange mechanisms and energetics of the most likely exchange path for Mo and W atoms (Fig. 3). The DFT model consisted of a triangular single-layer WS_2_ crystal with 45 W atoms and 103 S atoms, as shown in Figure S4 of the Supporting Information. A S vacancy, as the most probable point defect in the crystal^38^, was placed in the interior of the WS_2_ lattice in the upper plane of chalcogen atoms. The adsorption of a single Mo impurity atom was then tested on all possible sites of the crystal. The most stable adsorption configuration consists of a Mo atom adsorbed on top of an edge W atom in the vicinity of the S vacancy, which displaces the W atom downward. This configuration is depicted as the initial stage in Fig. 3. In stage 1, the Mo atom and one neighboring S atom diffuse toward the S vacancy to temporarily form antisite defects until they eventually reach the vacancy in stage 2. At this point, the S atom fills in the original S vacancy – thereby creating a new vacancy site in the upper plane of chalcogen atoms – and the Mo atom is positioned on top of a different W atom within the crystal. In stage 3, the S atom moves from the lower plane of chalcogen atoms to the upper plane of chalcogen atoms and the Mo atom moves downward into the plane of metal atoms, forcing the W atom to occupy the S vacancy site in the lower plane of chalcogen atoms. In the final stage, the W atom is expelled further toward the crystal edge to the metal site. It is important to note that the system is capable of overcoming the stage 2 energy barrier at the experimentally tested growth temperatures. Altogether, this process demonstrates a thermodynamically viable mechanism for the inward diffusion of Mo atoms accompanied by an outward diffusion of W atoms and S vacancies, turning a relatively defective WS_2_ crystal into a higher quality crystal with a Mo-rich core. This mechanism corroborates and helps to explain the S vacancy-Mo coupling in W-rich Mo_*x*_W_1−*x*_S_2_ reported by Azizi *et al*.^39^.

**Figure 4:**
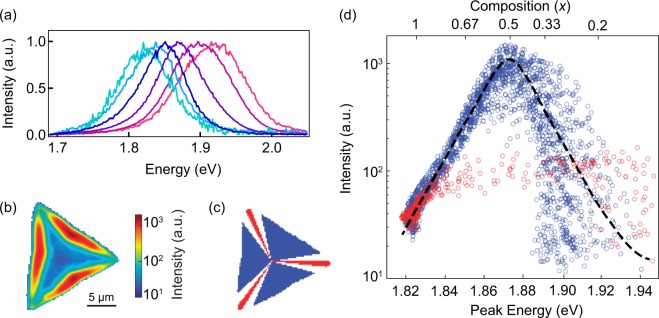
(**a**) Normalized PL spectral evolution from the crystal core (cyan, left-most spectrum) toward the edge (red, right-most spectrum). Spectra are individually fitted to double-Gaussian distributions and show a continuous change in peak emission energy corresponding to local alloy composition. (**b**) Spatial map of the photoluminescence (PL) intensity within the crystal shown in Fig. 1, plotted on a log scale. (**c**) Color-coded map of the crystal indicates PL data points extracted for the unstrained (blue) and strained (red) regions, as shown in (d). From the crystal center, all pixels of the crystal within 1.5 degrees of the symmetry lines connecting the center to the corners are colored red, indicating regions of greatest strain. All pixels greater than 6 degrees away from the symmetry lines are colored blue, indicating regions of least strain. (**d**) PL peak energy *vs*. intensity of the graded alloy crystal. Top scale shows the corresponding composition *x* determined from Raman measurements (we note that the scale is not linear due to non-linear relation between the PL peak energy and the composition). The blue(red) data points in the graph were taken from the blue(red) (*i*.*e*. unstrained(strained)) regions of panel (c). Dashed line is a guide for eye for the unstrained data, which mostly follows the average value of the blue data points.

The unique graded structure allows us now to investigate the material properties as a function of the composition within a single crystal, thus obtained under identical synthesis conditions. Here we focus on developing understanding of the PL quantum yield as a function of the Mo_*x*_W_1−*x*_X_2_ alloy composition. Figure 4a shows normalized position-dependent PL spectra that demonstrate a continuous transition from MoS_2_-like emission near the crystal core to WS_2_-like emission near the crystal edges. A good fit can be obtained for each emission spectrum, providing a reliable measurement of the PL peak intensity and energy. By comparing Raman and PL peak energy maps shown in Fig. 1a,b, respectively, we can determine correlation between the local crystal composition and corresponding PL energy, shown in Figure S5 of the Supporting Information.

Furthermore, composition-dependent PL intensity gives us an important insight into the relationship between PL quantum yield and the alloy composition. Although PL quantum yield specifically refers to the ratio between electron-hole pairs generated and photons radiated, we can make reasonably accurate relative comparisons from PL intensity fluctuations within a single measurement because the laser power density and acquisition time are constant, the absorbance for monolayer MoS_2_ and WS_2_ at 532 nm are approximately equal^40^, and the effects of exciton funneling are assumed to be negligible. Figure 4b maps the intensity of the PL peak determined by fitting individual PL spectra as a function of the position. By plotting the PL intensity along the lines that connect the crystal core to the triangle sides (sketched blue in Fig. 4c), it can be observed that PL intensity increases and decreases exponentially as a function of the PL energy, with the maximum PL intensity centered at ~1.87 eV (Fig. 4d). This result suggests that the maximum PL quantum yield in Mo_*x*_W_1−*x*_X_2_ system occurs for an alloy (in our case, for *x* ≈ 0.5) rather than for either of the pure extrema (MoS_2_ or WS_2_). In contrast to previous reports that have focused on either pure WS_2_ and MoS_2_^41^ or limited-range, Mo-rich Mo_*x*_W_1−*x*_Se_2_^42^, our results probe the PL quantum yield for nearly the entire Mo_*x*_W_1−*x*_S_2_ alloy spectrum.

Similar PL enhancement has been reported in ternary alloys of III–V semiconductors^43,44^. In those material systems, the enhanced radiative recombination has been attributed to reduced carrier mobility resulting in enhanced carrier localization. However, due to the large exciton binding energy of 2D TMDs^3^, the analogy between the two systems is not straight-forward. Instead, the PL enhancement could be the result of a decrease in concentration of non-radiative point-defect related deep levels in the alloy, as theoretically predicted^45^.

In addition, our results indicate that the PL quantum yield of Mo_*x*_W_1−*x*_X_2_ materials is also sensitive to strain. Figure 4d shows that the PL intensity is reduced by more than an order of magnitude for the same PL energy/crystal composition when measured from the crystal core toward the corners (sketched red in Fig. 4c). Generally, the strain accumulates at the corners due to the significant difference in temperature between growth conditions (825 °C for WS_2_ and 680 °C for MoS_2_) and room temperature as well as the difference in the coefficient of thermal expansion between TMDs and the substrate, SiO_2_^15,17,25,46–48^. In our crystals, this strain is evidenced in AFM height and phase maps (Supporting Figure S6). Given the low PL intensity in the strained regions at all compositions, we can conclude that the strain-dependent effects on PL quantum yield supersede the composition-dependent effects. Therefore, it is evident that strain must be well-controlled, whether through crystal transfer^49^, substrate choice^50^_,_ or other means, to control the luminescence yield regardless of crystal composition.

In conclusion, we have established the relationship between PL intensity and alloy composition (*x*) within single crystals of Mo_*x*_W_1−*x*_S_2_ graded alloys that span nearly the entire composition range. Our results show an exponential increase of the quantum yield with increased alloying, reaching the maximum value for equicompositional alloy Mo_0.5_W_0.5_S_2_ (*x* = 0.5), which is two orders of magnitude enhanced compared to the values for pure MoS_2_ (*x* = 1) and pure WS_2_ (*x* = 0). Such observation is consistent with theoretical prediction that alloying could suppress the deep in-gap level responsible for non-radiative recombination, and offers the venue for improving optical performance of TMD materials. Additionally, we demonstrated that the introduction of the strain leads to suppression of PL intensity for all the compositions of the alloy. The synthesis of graded alloys was achieved by diffusion-driven metal exchange in the pure TMD template crystals. Our DFT calculations reveal a thermodynamically favorable diffusion-exchange reaction path for controlled alloying, controlled by the sulfur vacancies. Our approach can be extended to other TMDs and future experiments to engineer the defect distribution by other means (e.g. laser, O_2_ plasma, annealing in air etc.) that would enable a higher degree of control over compositional tuning and patterning in a crystal. On-demand alloys such as these could be useful in several applications that require controlled, directional anisotropy of optical and/or electrical properties.

## Methods

### Sample Preparation

Mo_*x*_W_1−*x*_S_2_ alloys were grown in two subsequent CVD steps at ambient pressure using Ar as a carrier gas. A bare Si wafer substrate with a 300-nm thick SiO_2_ layer was suspended above an alumina boat containing the metal precursor powder at the center of the CVD furnace. Sulfur powder (Sigma-Aldrich, 99.5%), the chalcogen precursor, was placed in another alumina boat and was initially outside of the furnace, at the upstream end of the 1-inch quartz tube. Once the furnace reached the growth temperature, the sulfur was introduced to the upstream of the furnace using magnets to reach approximately 200 °C. In the first growth step, WS_2_ crystals were grown at 825 °C for 30 min using tungsten trioxide (WO_3_, Sigma-Aldrich, 99.9%) mixed with sodium chloride (NaCl) in a 9:2 mass ratio as the metal precursor with an Ar flow rate of 50 sccm. In the second growth step, the metal precursor was replaced with molybdenum trioxide (MoO_3_, Sigma-Aldrich, 99.5%), the growth temperature was reduced to 680 °C and Ar flow rate to 25 sccm.

Exfoliated crystals were produced from bulk samples by mechanical exfoliation.

### Sample Characterization

Optical images were recorded using a Leica DFC 450 C microscope. AFM images were recorded with a Bruker Dimension Fastscan AFM using a tapping mode. Raman and PL measurements were performed on WITec Alpha 300 R and Horiba Jobin Yvon LabRAM HR800 confocal Raman microscopes under ambient conditions at room temperature. A 532 nm laser was focused onto the sample using a 100× objective resulting in a spot size of ~1 µm in diameter. 1800 and 600 lines/mm grating were used in Raman and PL measurements, respectively.

### DFT Calculations

Computational results utilize density functional theory implemented in Vienna ab-initio Simulation Package^51,52^. The plane-wave basis with the cut-off energy of 400 eV and the generalized gradient approximation in the Perdew-Burke-Ernzerhof (PBE) format^53^ with the projector-augmented wave method^54^ were employed in all calculations. The structural relaxations were carried out until the Hellmann-Feynman force on each atom is less than 0.01 eV/Å, and the energy convergence criterion was set to 10^−5^ eV. Gamma-point-only *k* sampling is adopted for calculations.

## Electronic supplementary material

Supplementary Material
