# Supplementary material for: Two-Dimensional MoxW1−xS2 Graded Alloys: Growth and Optical Properties
Source: Sci Rep. 2018 Aug 27;8:12889. doi: 10.1038/s41598-018-31220-z (PMC6110786; doi:10.1038/s41598-018-31220-z)
Supplement: Supplementary file 1 — Supplementary Material [file 41598_2018_31220_MOESM1_ESM.pdf]

Supporting Information

Two-Dimensional $Mo_xW_{1-x}S_2$ Graded Alloys: Growth and Optical Properties

*Kevin Bogaert[1,2], Song Liu[2], Tao Liu[2,3], Na Guo[2,3], Chun Zhang[2,3,4], Silvija Gradečak[1,5], Slaven Garaj[2,3,6]**

[1]Department of Materials Science and Engineering, Massachusetts Institute of Technology, 77 Massachusetts Avenue, Cambridge, MA 02139, USA

[2]Centre for Advanced 2D Materials and Graphene Research Centre, National University of Singapore, 6 Science Drive 2, 117546, Singapore, Singapore

[3]Department of Physics, National University of Singapore, 2 Science Drive 3, 117542, Singapore, Singapore

[4]Department of Chemistry, National University of Singapore, 3 Science Drive 3, 117543, Singapore, Singapore

[5]Low Energy Electronic Systems Interdisciplinary Research Group, Singapore-MIT Alliance in Research and Technology, 138602, Singapore, Singapore

[6]Department of Biomedical Engineering, National University of Singapore, 9 Engineering Drive 1, 117575, Singapore, Singapore

Figure S1 shows an optical image and atomic force microscopy (AFM) height map of the $Mo_xW_{1-x}S_2$ crystal discussed in Figures 1 and 4 of the main text. Despite the significant difference in composition between the crystal core ($x \approx 1$) and the edge ($x \approx 0$), the crystal lacks a

definitive interface in the optical image (Figure S1(a)) due to the gradual change in its composition. The AFM image in Figure S1b demonstrates that the sample has a uniform thickness.

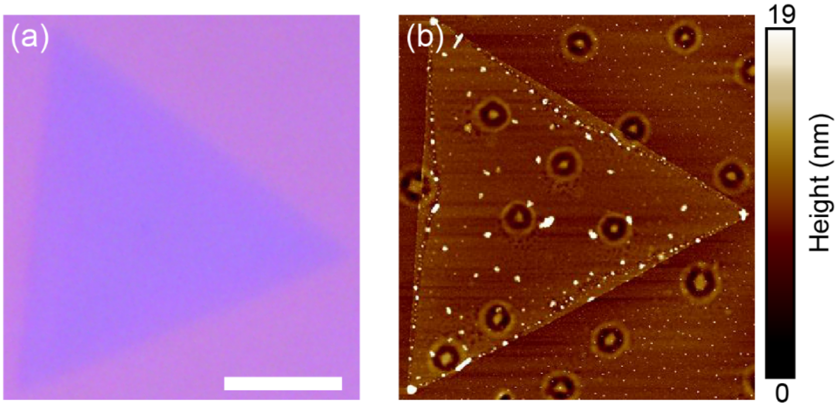

Figure S1. (a) Optical microscopy and (b) AFM height map of graded alloy crystal shown in Figure 1. Scale bar is 5 μm.

Figure S2 provides photoluminescence (PL) and Raman data corresponding to another example of crystals with graded alloying produced by the same method described in the main text for Figures 1 and 4. All measurements yield similar results, demonstrating the reproducibility of our technique. The high intensity near 350 cm^{-1} and 420 cm^{-1} at 0 μm in Figure S2c is attributed to a small multi-layer of pure WS_2 on top of the alloyed crystal. This gives further credence to the idea that alloying is happening *via* lateral diffusion as opposed to vertical incorporation.

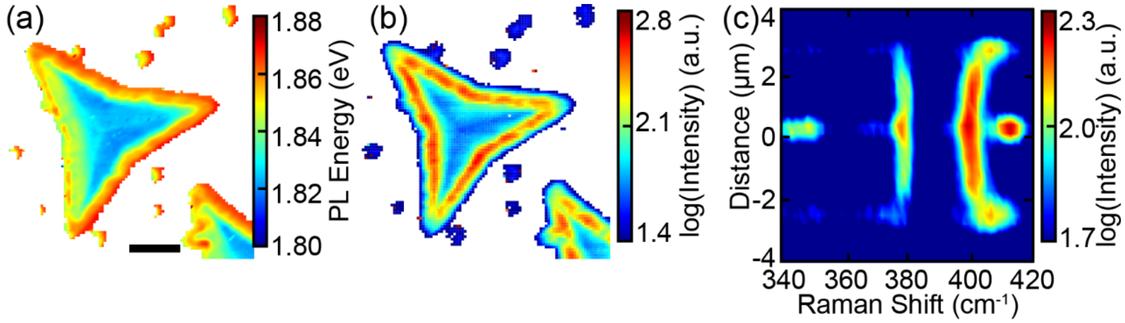

Figure S2. (a) PL energy and (b) intensity, and (c) Raman line scan of $Mo_xW_{1-x}S_2$ graded alloy crystals from a different growth than the crystal shown in Figures 1 and 4 of the main text. Scale bar is 5 μm.

Figure S3 shows distribution of point defects in a WS_2 crystal that was grown with the assistance of NaCl. The Raman spectrum labels the LA(M) and A vibrational modes of WS_2 at ~174 and ~419 cm^{-1}, respectively. The ratio of intensities between these two vibrational modes is mapped in the inset and has been reported[1] to correlate with the spatial density of point defects in the crystal. The spatial distribution of the intensity ratio provided in Figure S3 correlates strongly with the Mo distribution in the final graded alloy crystal shown in Figure 1 of the main text.

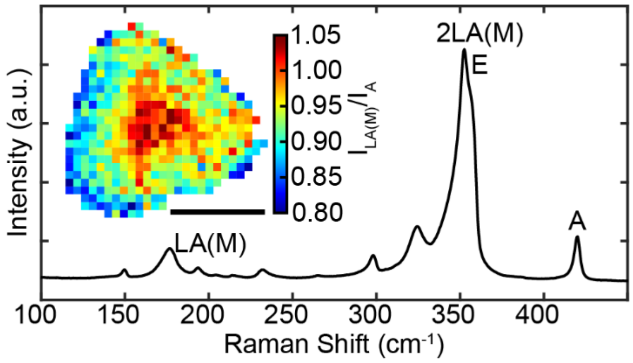

Figure S3. Averaged Raman spectrum of a WS_2 crystal grown using NaCl. Inset maps the ratio between intensities of LA(M) (174 cm^{-1}) and A (415 cm^{-1}) vibrational modes showing a graded distribution of defects within the crystal with the highest concentration in the center. Scale bar is 5 μm.

Figure S4 provides the full crystal configuration used in the density functional theory (DFT) calculations discussed in the main text and shown in Figure 3. The crystal contains 103 S atoms, 45 W atoms, 1 Mo adatom, and 1 S vacancy. The Mo adatom is positioned on top of an edge W atom and the S vacancy is in the interior of the crystal, near the Mo adatom, and in the upper plane of chalcogen atoms. This crystal is a simplified model of what is expected to occur during the second step of the two-step growth process. The S vacancy in the interior is a consequence of a defect-prone WS_2 growth step, as demonstrated in Figure S3. The Mo adatom is introduced during the subsequent MoS_2 growth step.

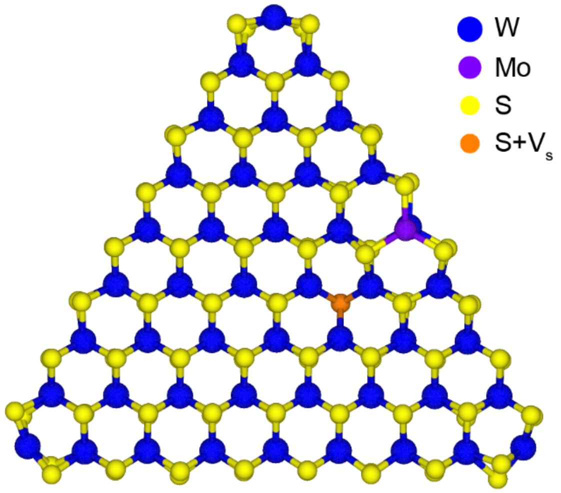

Figure S4. Monolayer WS_2 crystal configuration used for DFT calculations discussed in the main text and shown in Figure 3. The crystal contains one S vacancy (orange) and one Mo adatom (purple).

Figure S5 correlates the position of the Raman A vibrational mode from Figure 1a with the PL peak energy from Figure 1b collected on the same crystal using the same microscope settings. The results indicate a strong correlation between these two measurements, suggesting that the established relationship[2] between alloy composition and the Raman A position can be extended to PL peak energy. This correlation was used to determine the alloy composition with the maximum PL quantum yield shown in Figure 4b of the main text.

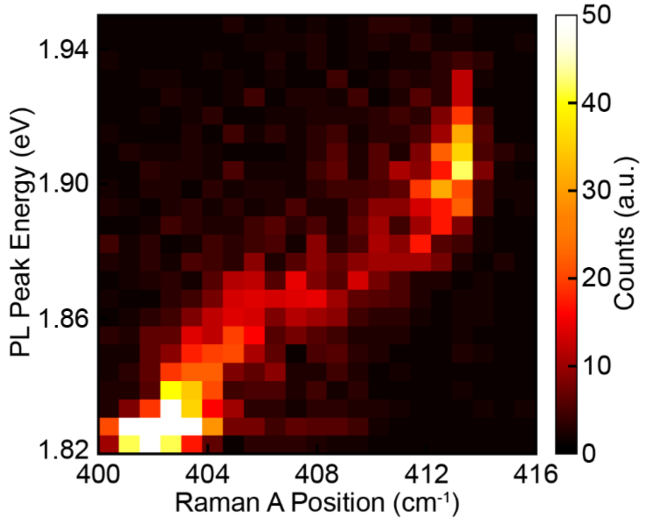

Figure S5. Heat map correlating the Raman position of the A vibrational mode and the PL peak energy from a single $Mo_xW_{1-x}S_2$ graded alloy crystal, using the data represented in Figure 1a and b, respectively.

The PL intensity variations presented in Figure 4b of the main text can be directly correlated to the strain distribution determined using AFM, as shown in Figure S6. Although the AFM height map in Figure S6a demonstrates uniform crystal thickness, the phase maps in Figure S6b and S6c show increased contrast along the lines spanning from the crystal core to the corners. These variations in the phase contrast can be attributed to increased strain and correlate with decreased PL intensity. Although the entire crystal experiences some degree of strain resulting from the difference in the thermal coefficient of expansion between the substrate and 2D TMD crystal, the strain is concentrated along the lines connecting the crystal core and corners. Similar correlation between strain and optical properties along the lines to the crystal corners has been previously observed in single crystal TMDs, sometimes leading to intra-grain boundaries or highly defective regions[3–8].

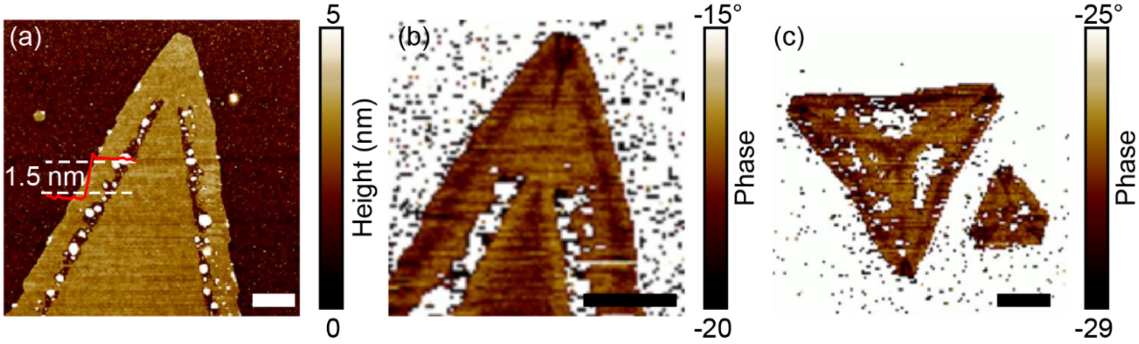

Figure S6. (a) AFM height and (b, c) phase maps for graded alloy crystals. Inset in (a) is height line scan. Corner-to-center lines show distinct features attributable to strain in phase maps (b, c) but not in height map (a). White regions within the crystals correspond to areas of high surface roughness attributable to crystal oxidation and/or adsorbed species. Scale bars are 1 μm.

References:

1. McCreary, A. *et al.* Distinct photoluminescence and Raman spectroscopy signatures for

identifying highly crystalline WS2 monolayers produced by different growth methods. *J. Mater. Res.* **31,** 931–944 (2016).

2. Chen, Y. *et al.* Composition-dependent Raman modes of Mo(1-x)W(x)S2 monolayer alloys. *Nanoscale* **6,** 2833–2839 (2014).

3. Lin, Z. *et al.* Facile synthesis of MoS2 and MoxW1-xS2 triangular monolayers. *APL Mater.* **2,** 92514 (2014).

4. Bao, W. *et al.* Visualizing nanoscale excitonic relaxation properties of disordered edges and grain boundaries in monolayer molybdenum disulfide. *Nat. Commun.* **6,** 7993 (2015).

5. Alharbi, A. & Shahrjerdi, D. Electronic properties of monolayer tungsten disulfide grown by chemical vapor deposition. *Appl. Phys. Lett.* **109,** 193502 (2016).

6. Meng, L. *et al.* Two dimensional WS2 lateral heterojunctions by strain modulation. *Appl. Phys. Lett.* **108,** 263104 (2016).

7. McCreary, K. M. *et al.* The Effect of Preparation Conditions on Raman and Photoluminescence of Monolayer WS2. *Sci. Rep.* **6,** 35154 (2016).

8. Bogaert, K. *et al.* Diffusion-Mediated Synthesis of MoS2/WS2 Lateral Heterostructures. *Nano Lett.* **16,** 5129–5134 (2016).
